# The Epidome - a species-specific approach to assess the population structure and heterogeneity of *Staphylococcus epidermidis* colonization and infection

**DOI:** 10.1186/s12866-020-02041-w

**Published:** 2020-11-26

**Authors:** Amalie Katrine Rendboe, Thor Bech Johannesen, Anna Cäcilia Ingham, Emeli Månsson, Søren Iversen, Sharmin Baig, Sofie Edslev, Jørgen Skov Jensen, Bo Söderquist, Paal Skytt Andersen, Marc Stegger

**Affiliations:** 1 Department of Bacteria, Parasites and Fungi, Statens Serum Institut, Copenhagen, Denmark; 2 School of Medical Sciences, Faculty of Medicine and Health, Örebro University, Örebro, Sweden; 3 Centre for Clinical Research, Hospital of Västmanland, Region Västmanland – Uppsala University, Västerås, Sweden

**Keywords:** Targeted sequencing, *Staphylococcus epidermidis*, Sequencing, Population structure, Heterogeneity, Population dynamics, Clonal lineages, *S. epidermidis*, Amplicon sequence variant, Species-specific classification

## Abstract

**Background:**

Although generally known as a human commensal, *Staphylococcus epidermidis* is also an opportunistic pathogen that can cause nosocomial infections related to foreign body materials and immunocompromized patients. Infections are often caused by multidrug-resistant (MDR) lineages that are difficult and costly to treat, and can have a major adverse impact on patients’ quality of life. Heterogeneity is a common phenomenon in both carriage and infection, but present methodology for detection of this is laborious or expensive.

In this study, we present a culture-independent method, labelled Epidome, based on an amplicon sequencing-approach to deliver information beyond species level on primary samples and to elucidate clonality, population structure and temporal stability or niche selection of *S. epidermidis* communities.

**Results:**

Based on an assessment of > 800 genes from the *S. epidermidis* core genome, we identified genes with variable regions, which in combination facilitated the differentiation of phylogenetic clusters observed in silico*,* and allowed classification down to lineage level. A duplex PCR, combined with an amplicon sequencing protocol, and a downstream analysis pipeline were designed to provide subspecies information from primary samples. Additionally, a probe-based qPCR was designed to provide valuable absolute abundance quantification of *S. epidermidis*. The approach was validated on isolates representing skin commensals and on genomic mock communities with a sensitivity of < 10 copies/μL. The method was furthermore applied to a sample set of primary skin and nasal samples, revealing a high degree of heterogeneity in the *S. epidermidis* populations. Additionally, the qPCR showed a high degree of variation in absolute abundance of *S. epidermidis*.

**Conclusions:**

The Epidome method is designed for use on primary samples to obtain important information on *S. epidermidis* abundance and diversity beyond species-level to answer questions regarding the emergence and dissemination of nosocomial lineages, investigating clonality of *S. epidermidis* communities, population dynamics, and niche selection. Our targeted-sequencing method allows rapid differentiation and identification of clinically important nosocomial lineages in low-biomass samples such as skin samples.

**Supplementary Information:**

The online version contains supplementary material available at 10.1186/s12866-020-02041-w.

## Background

Staphylococci constitute an important group of human skin and mucosal commensals [1, 2], where coagulase-negative staphylococci (CoNS) generally are regarded to have a benign or symbiotic relationship with their hosts [3]. However, certain species are opportunistic human pathogens and play an important role in especially nosocomial infections [4, 5]. Among the CoNS, *Staphylococcus epidermidis* has recently gained much attention as a significant nosocomial pathogen associated with foreign body infections such as prosthetic joint infections (PJIs) [6, 7]. Furthermore, *S. epidermidis* is a common causative microorganism in infections in immunocompromized patients, including preterm infants and patients with hematological malignancies [4, 8, 9]. These infections result in large economic costs for society [10, 11], and can have major adverse impact on patients’ quality of life [12].

A large percentage of hospital *S. epidermidis* isolates are methicillin-resistant (MRSE), and especially multidrug-resistant *S. epidermidis* (MDRSE) lineages, such as sequence type (ST)2, ST5, ST23, and ST215, are spreading both locally and globally in hospital environments [6, 8, 13–17]. Identification of *S. epidermidis* as an invasive infectious pathogen is not trivial and distinguishing contamination from true infection can be challenging, and research suggests that high sub-species heterogeneity is common in healthy carriage [18, 19], but also in patients suffering from PJIs and atopic dermatitis [20, 21]. This observation is in contrast to the nature of *Staphylococcus aureus* communities that are known to be highly clonal [22–24].

Methods to elucidate clonality, population dynamics and temporal stability or niche selection of *S. epidermidis* communities due to antimicrobial prophylactic treatments currently include laborious culturing and characterization of multiple isolates or application of expensive metagenomics sequencing. In this paper we present the Epidome, a culture-independent method based on amplicon sequencing of two *S. epidermidis*-specific target genes. Similar amplicon-based methods exist to identify staphylococci at genus level [25], and to distinguish between various CoNS [26–28].

The Epidome method is designed for use on primary samples to obtain information on *S. epidermidis* abundance and diversity beyond species-level to answer important questions regarding the emergence and dissemination of nosocomial lineages. Here, we demonstrate our method on a sample set comprising nasal and skin swab samples from healthy community-dwelling adults.

## Results

In order to establish the Epidome method, we designed and tested the specificity of primers for amplicon sequencing of two *S. epidermidis* specific targets. Furthermore, we set up a publicly available analysis pipeline for sequencing quality control, amplicon sequence variant (ASV) inference, and taxonomic classification (https://github.com/ssi-dk/epidome/). For the latter, we built two target-specific databases. A qPCR-based approach to complement the amplicon sequencing was also implemented. Below, we present our method and demonstrate the use of the analysis pipeline on an exemplary data set from human nasal and skin samples.

### Population structure and Epidome gene target diversity

**Fig. 1:**
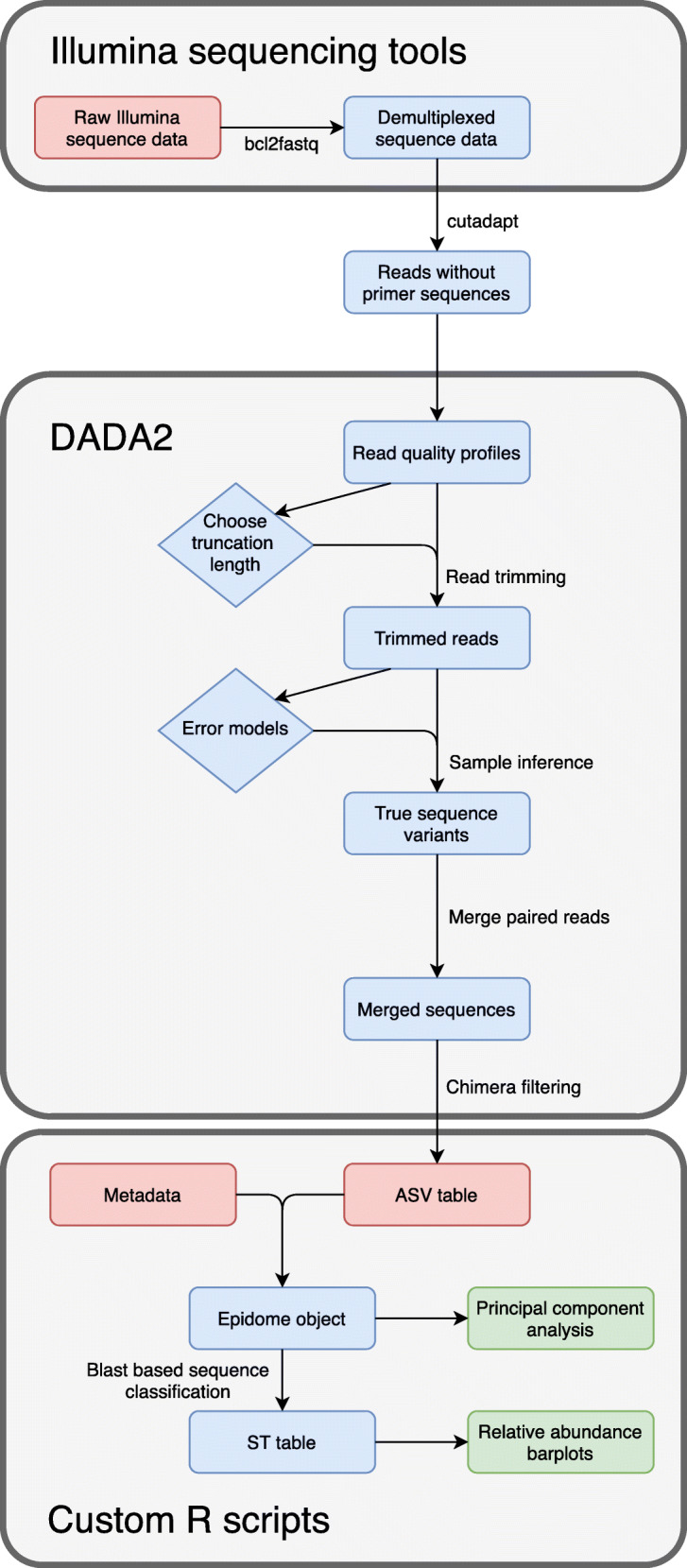
A schematic overview of the Epidome analysis pipeline from sequence reads to classification and figure outputs

To infer clonal lineages of *S. epidermidis*, we investigated the population structure of the *S. epidermidis* population (*n* = 842) based on 86,139 SNPs identified in a 39% core (1,033,603 bp) using the high-quality requirements for retaining positions across the collection containing both assembled RefSeq genomes and data from 289 Swedish invasive and colonizing isolates (Fig. 1a). Definition of clonal groups by hierarchical clustering on pairwise SNP-dissimilarities defined 130 clonal groups.

### Primer design for amplicon sequencing

Based on an assessment of the core genome among 50 *S. epidermidis* isolates representing the diversity observed in the core phylogeny, we identified a total of 1,900 candidate genes. When analyzing these for requirements as suitable targets for a species-specific amplicon-based approach (highly variable region flanked by conserved primer binding sites) we identified a total of 1088 primer pairs positioned in 89 different genes. Presence of the top candidate genes was then assessed across the entire population of 842 *S. epidermidis* genome sequences. Here, two targets (*yycH* and *g216*, locus tags SE_0020 and SE_0191) in *S. epidermidis* isolate ATCC 12228 (GenBank Accession ID AE015929) were chosen for a duplex PCR that in combination resembled the phylogenetic clustering observed on the basis of core genome SNPs (Fig. 1b). The *g216* gene was the primary target as it offered the highest resolution of the population as a whole, whereas the combination with *yycH* allowed key endemic nosocomial lineages to be uniquely identified in silico.

### Specificity testing and reproducibility of the Epidome method

**Fig. 2:**
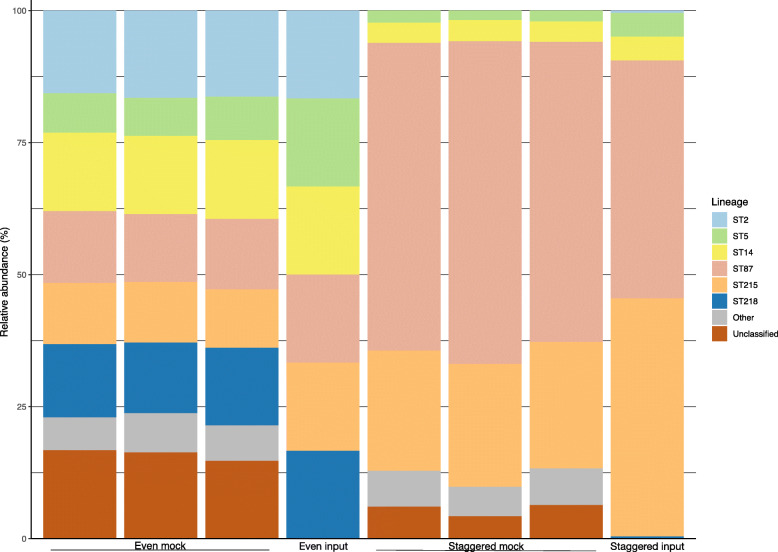
Sequencing of *Staphylococcus epidermidis* even and staggered mock communities. Data represent results of the amplicon sequencing of technical triplicates consisting of even mock communities comprised of 10,000 copies of *S. epidermidis* ST2, ST5, ST14, ST87, ST215, and ST218, and staggered mock communities with 10,000 copies of ST215 and ST87, 1000 copies of ST5 and ST14 and 100 copies of ST2 and ST218. The data is shown as relative abundance per sample. Category ‘Other’ includes clonal groups detected, but not expected in the mock samples

Using DNA from *S. epidermidis* isolates (*n* = 26) representing 15 clonal groups including major hospital-associated lineages such as ST2, ST5, and ST215 as well as from other staphylococcal species (*n* = 27) and other common skin commensals (*n* = 21, see Supplementary Table 1), both primer sets were tested in separate PCR reactions. Here, the *g216* PCR was found to be *S. epidermidis* specific, whereas the *yycH* PCR resulted in amplification of four non-*S. epidermidis* species out of the 48 tested *(S. equorum, S. haemolyticus,* S*. pseudintermedius*, and *S. argenteus*). Importantly, the sequences of these four species were distinct from *S. epidermidis* and were excluded from downstream analyses. The Epidome duplex PCR containing both the *g126* and *yycH* primers was run under the same conditions as the singleplex reactions. To evaluate the capabilities of the multiplex PCR, sequencing and downstream analyses, the method was applied in triplicates of both even- and staggered genomic mock communities with six STs represented (Fig. 2). Even when present in low abundance down to 100 copies/μL, the STs were successfully detected with a high level of reproducibility for technical replicates (Fig. 2). However, for the staggered mocks a reliable detection sensitivity of ~ 1% was observed (Fig. 2), as illustrated by the lack of detection of the ST2 and ST218 in these with the present sequencing depth. In the mocks, ASVs classified as Others include ST307, ST252 and sequences matching isolates with novel sequence types. Due to the expected rate of read errors in Illumina sequencing, some ASVs matched with these sequence types despite not being present in the sample. ST307 was the most prominent of these misclassified types and represented up to 2% of reads from a sample, which indicates that classification of ASVs found at or below this level in a sample should be considered unreliable. Unclassified ASVs are sequences which had less than 98.5% similarity with any sequence in the database.

### Amplicon sequence-preprocessing and taxonomic classification

For each of the two target genes, we set up a bioinformatic analysis pipeline comprising primer trimming, quality filtering and truncation of forward and reverse reads, Illumina error profile estimation, and inference of ASVs following amplicon sequencing. For each of the two amplicon targets, a custom blast database was set up containing all unique sequences found in the collection of 842 *S. epidermidis* genomes. Taxonomic classification was based on a combination of sequences from both target genes (see Methods section).

Twenty-two primary samples, collected from skin (*n* = 11) and anterior nares (n = 11) of healthy humans, were sequenced in technical duplicates. We obtained on average 14,927 initial reads of *g216* gene sequences per sample. On average, 10,806 reads per sample (71% of the initial reads) passed quality filtering and had a read length exceeding the specified minimum. Of these reads, on average 10,482 reads per sample (99%) were non-chimeric reads. The read count of the two blank extraction controls after all filtering steps were 36 and 408, respectively.

*YycH* gene sequencing yielded on average 46,915 initial reads per sample. On average, 30,406 reads per sample (63% of the initial reads) passed quality and length filtering. Thereof, on average 28,999 reads per sample (98%) were non-chimeric reads. The two blank extraction controls had 707 and 1441 reads after all filtering steps, respectively. Read counts revealed an overall even distribution of numbers of Illumina reads for the *yycH* and *g216* targets across all samples (Pearson’s correlation coefficient of 0.94, see Supplementary Fig. 1), illustrating the linear co-amplification across all samples with a higher efficiency of the *yycH* target.

To investigate whether any difference in read counts between samples were related to the detected richness of *S. epidermidis*, we generated rarefaction curves to display the number of observed ASVs over the library size per sample after quality filtering, see Supplementary Fig. 2A and 2B for *g216* and *yycH*, respectively. A read count that was low (defined here as < 5000) in both technical replicates of a sample (e.g. P06_skin_1 (3885 reads) and P06_skin_2 (1771 reads)) indicated a low abundance of *S. epidermidis* in this sample (Supplementary Fig. 2A). This indication was reinforced in those samples where the rarefaction curve reached a plateau with regards to the number of observed ASVs despite a low read count. In the few samples where the read count was higher in one replicate than in the other, and where the replicate with the lower read count does not reach a plateau of observed ASVs (e.g. P03_skin_2 (11 ASVs in 3143 reads) vs P03_skin_1 (20 ASVs in 5827 reads)), the low read count is likely attributable to technical differences (Supplementary Fig. 2A). Overall, on the analyzed sample collection, 5000 filtered reads/sample seems sufficient to describe the richness of *S. epidermidis* on the skin and in the nares.

### *S. epidermidis* heterogeneity in primary human skin and nasal samples

**Fig. 3:**
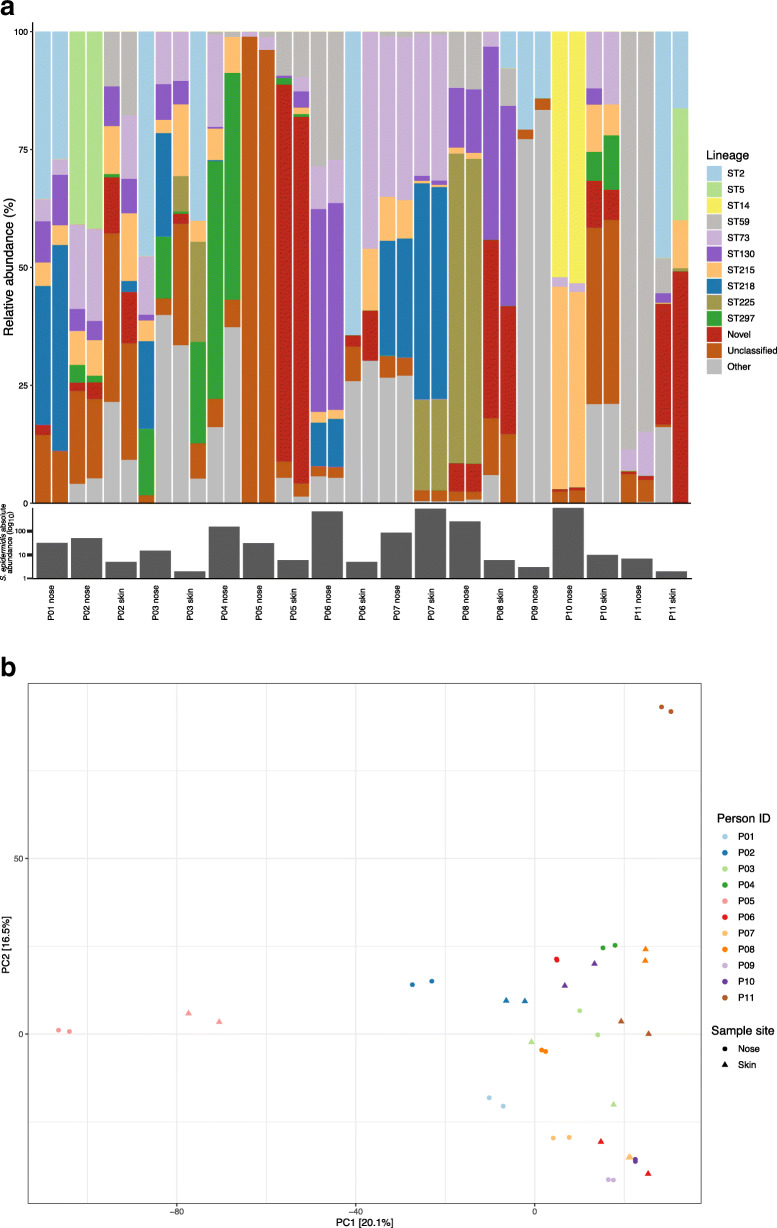
Carriage heterogeneity of *Staphylococcus epidermidis* on human skin and in nares. Results of the Epidome methodology with technical replicates on skin and anterior nasal samples from 11 individuals. **a** Relative abundance of detected clonal groups identified among the primary samples. The lineage legend highlight the 10 most prevalent clonal groups found across all samples. Reads not mapping to any sequences in the constructed reference database (Novel), mapping to sequences with no known ST (Unclassified), or to other characterized lineages (Other) are similarly presented. The median of the absolute abundance of *S. epidermidis* in the two primary samples using qPCR of the *g216* gene target in triplicates are depicted below samples on a log_10_ scale. **b** Assessment of the reproducibility of the Epidome method on primary samples and between-sample diversity using a principal component analysis depicting the beta-diversity on the relative abundances of ASVs based on a two-sampling approach per site per individual

To validate the method on primary samples, it was applied to 22 swab samples from paired human skin (*n* = 11) and anterior nares (n = 11). We found that 20/22 (91%) of all primary samples were positive for *S. epidermidis* and that the samples generally showed a high degree of inter-sample heterogeneity regarding the presence of *S. epidermidis* lineages (Fig. 3). Samples were found to contain between two and 8 different clonal groups with an mean of five in each sample.

The majority of sequencing reads (76%) were distributed within 10 different clonal groups (ST2, ST5, ST14, ST59, ST73, ST87, ST130, ST215, ST218 and ST225). A subset of reads (5%) was assigned to other clonal groups while the rest of the reads either mapped to *S. epidermidis* sequences in the database with no known ST (Unclassified, 5%) or did not map to any sequences in the constructed reference database containing all presently available genome sequenced isolates of *S. epidermidis* (Novel, 13%).

We assessed the beta-diversity between the primary samples using a PCA which showed a high level of beta-diversity across all samples but also that the nose and skin had overlapping populations of *S. epidermidis*, see Supplementary Fig. 3. Importantly, pairwise clustering of technical duplicates of primary samples was observed, depicting the reproducibility of the Epidome methodology (Fig. 3b).

### Absolute abundance of *S. epidermidis* assessed by qPCR

To extend the insights that compositional data generated by amplicon sequencing provides, we developed a complementing qPCR approach to measure absolute abundance. In the designed qPCR setup, we used the *g216* target gene as a basis for which we successfully designed an MGB probe for qPCR detection and quantification of mixed *S. epidermidis* populations. Our analyses on serial 10-fold dilutions of *g216* amplicons revealed an R^2^ of 0.99 across seven log_10_ dilutions with an efficiency of 70% and a limit of detection of < 10 copies/μL. Despite the low efficiency, the technical replicates showed excellent reproducibility (Supplementary Table 2). The qPCR was applied to our 22 primary samples in technical triplicates. *S. epidermidis* was detected in all but two samples with quantities per sample ranging from 2 to 962 copies/μL (Fig. 3a). Nasal samples generally exhibited a higher quantity of *S. epidermidis* than skin samples. Non-template controls were negative by qPCR. For samples with low absolute abundance, a higher degree of variation in the technical replicates was observed.

## Discussion

We designed and validated a novel two-step method for identification and quantification of *S. epidermidis* from primary samples using targeted amplicon sequencing and qPCR.

While 16S rRNA gene sequencing is the most widely used tool for studying microbial communities, this method does not generally provide comprehensive information beyond species level. The approach described here, labelled the Epidome, is an alternative culture-independent method that rapidly provides high taxonomic resolution beyond species-level of heterogeneous populations of *S. epidermidis* without the cost associated with metagenomics and without being as time-consuming and laborious as cultivation-based methods.

Designing targets that unambiguously distinguish between all major lineages is however a major challenge as *S. epidermidis* is highly diverse and recombination is frequent in this species [15, 29]. Our approach was to include all available *S. epidermidis* genome sequences available in the public repository and to have a specific focus on resolving major *S. epidermidis* groups that are known to be the main causes of hospital-acquired infections; ST2, ST5, ST23, and ST215. Our method has proven successful in detecting and distinguishing between these selected groups as well as in providing insight into the overall *S. epidermidis* diversity. Thus we believe that the method will be a useful tool to study colonization rates, population dynamics and temporal stability or niche selection due to antimicrobial prophylactic treatments in relation to infections of this increasingly important nosocomial pathogen.

Despite that a multitarget approach would have provided more discriminatory power, we used a two-gene target approach to distinguish between our defined *S. epidermidis* clonal groups to allow assignment to distinct branches of the *S. epidermidis* population while reducing the computation uncertainly of matching multiple, potential novel, targets in a heterogeneic sample material. In silico analysis against NCBI’s nucleotide collection (nr/nt) database showed no off-target amplification for any of the two primer sets. The *g216* target was specific for *S. epidermidis* while the *yycH* target resulted in sporadic non-*S. epidermidis*-specific amplification. The amplicons of these other species were highly dissimilar compared to *S. epidermidis* making downstream exclusion easily feasible, and thus not a major limitation of the methodology. The *g216* gene was selected as the qPCR target since it displayed high specificity for *S. epidermidis.* The qPCR showed sufficiently low detection limits and allowed highly accurate detection and quantification of mixed *S. epidermidis* populations.

The Epidome method allowed differentiation of *S. epidermidis* clonal groups in primary samples but also revealed a high number of novel sequences from the investigated primary samples that did not map to the reference databases. This suggests that either the diversity of naturally occurring *S. epidermidis* on the skin and in the nares of healthy individuals is higher than the sum of all published *S. epidermidis* genomes or that the high frequency of homologues recombination in *S. epidermidis* results in gene target combinations that are novel. In the nosocomial lineages, we observe that the targets are conserved, even though the genomic data originates from both temporally and spatially diverse collections.

**Fig. 4:**
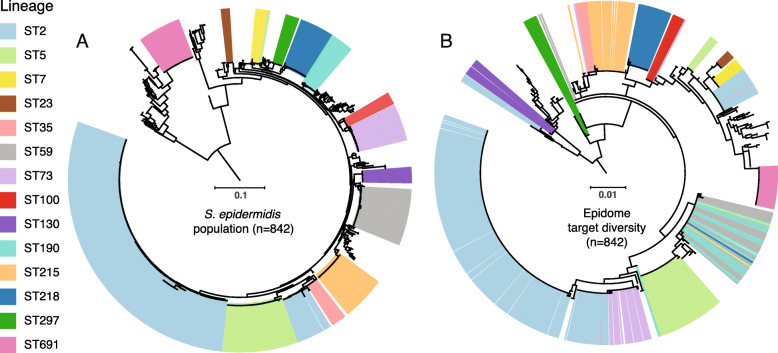
Phylogenetic clustering of the *Staphylococcus epidermidis* population and Epidome gene targets. **a** Unrooted phylogeny of 842 *S. epidermidis* genomes based on 86,139 core genome SNPs. Major lineages as well as clinical important multidrug-resistant lineages are highlighted. **b** Unrooted phylogeny of the concatenated sequence of the Epidome target genes across the *S. epidermidis* population depicting the lineage origin of all targets. Both phylogenies were annotated using iTol. Scale bars indicate substitutions per site

Looking at the beta-diversity within the primary samples from skin and nares, our data indicates that the *S. epidermidis* community on the skin is more homogenous than in the nares, although a dedicated study is needed to validate this finding. In terms of reproducibility, our technical replicates of the mock communities and primary samples (Fig. 4a and Fig. 3b, respectively) show a high level of congruence. We believe that these results combined confirm that this method has potential as a tool to study in detail the population dynamics and diversity of *S. epidermidis*.

The accuracy of amplicon-sequencing based identification methods is naturally dependent on the underlying database used for classification. Importantly, the database used here, while extensive, is not expected to capture the entire diversity of the *S. epidermidis* population. Exactly how much of the diversity of *S. epidermidis* that still lack whole-genome sequenced data is hard to estimate. However, our observation that ~ 10% of *S. epidermidis* ASVs from nose and skin samples were labelled as Unclassified (not assignable to a single clonal group) or Novel (ASVs not matching any known variants in the database) provides an indication of the minimum level of unknown *S. epidermidis* variants we can expect to find in these environments. Our method thus also highlights the need to increase sequencing efforts to provide the taxonomic information necessary to classify the diverse *S. epidermidis* clonal groups which we now are able to identify. Other methods that rely on DNA fingerprinting such as PFGE or AFLP, would inherently be highly complex to analyze due to the heterogeneity observed in *S. epidermidis* populations and with limited ability to assign distinct lineages. Contrary, genome sequencing will naturally provide much more information on individual isolates in a clinical setting, but for research studies aimed at understanding heterogeneity and population dynamics, the Epidome approach will provide unparalleled insight.

In this approach we assigned all unique ASV sequences in our database to clonal groups. Given the incompleteness of the database, yet undescribed *S. epidermidis* ASV sequences may match equally well to multiple similar reference sequences, bearing a risk for misassignment. However, it is most likely that those sequences would remain Novel, or be misassigned to a closely related clonal group.

It should also be noted that the whole-genome sequencing data currently available on *S. epidermidis* is largely dominated by isolates from human nasal and skin samples as well as infections – particularly infections from indwelling medical devices. Other niches may be populated by a very different population of *S. epidermidis*, in which case the accuracy of sequence type classification will inevitably be lowered. Regardless of the completeness of the database, unbiased analysis based on ASVs identified with DADA2 can always be performed and can provide valuable information on the stability of *S. epidermidis* communities, temporal changes in composition, and similarities between sampling sites.

## Conclusions

We successfully designed a PCR- and amplicon sequencing-based approach - the Epidome – to provide insight into *S. epidermidis* quantity and diversity beyond species-level. Our targeted sequencing method allows reproducible identification of important epidemic nosocomial lineages in primary samples even when present in low abundance. To strengthen interpretation of the sequencing data we designed and validated a qPCR that provides an important quantification of *S. epidermidis* population in primary samples. Our analyses on primary samples also highlight the heterogeneity of *S. epidermidis* found on the skin and in the nose of healthy individuals. Importantly, our analysis pipeline, underlying database, and supporting documentation for investigating the population of *S. epidermidis* are publicly available.

Using the Epidome, a number of questions can be answered without the need for laborious and biased cultivation and without using costly methods such as metagenomics. We believe that the approach shows great potential especially for studying the important issues of heterogeneity of *S. epidermidis* communities, population dynamics and temporal stability or niche selection due to antimicrobial prophylaxis or treatments.

## Methods

To investigate the diversity of *S. epidermidis* at various sampling sites such as the skin and nose, we aimed at designing a simple quantitative approach using parallel sequencing as offered by next generation sequencing technology on species-specific targets that represent the population diversity of the species, see Fig. 1. The method was designed and validated on primary samples representing body sites of colonization.

### Population structure of *S. epidermidis*

The dataset used for identification of suitable amplicon targets consisted of all 553 *S. epidermidis* genomic assemblies obtained from the NCBI’s Reference Sequence Database (RefSeq, https://www.ncbi.nlm.nih.gov/refseq/, accessed September 2019) and 289 SPAdes [30] assembled genomes from a recent Swedish study of *S. epidermidis* and PJIs [31]. Importantly, the Swedish collection included ST215/ST434 MDRSE isolates but also a diverse population of antibiotic susceptible isolates from healthy carriers.

To evaluate the population structure of *S. epidermidis*, single nucleotide polymorphisms (SNPs) from the core genome across all 842 genomes were obtained with the Northern Arizona SNP Pipeline (NASP) [32]. Here, paired-end Illumina reads from the 289 isolates from the Swedish collection and genomic assemblies from the 553 RefSeq isolates were mapped to the reference chromosome of *S. epidermidis* isolate ATCC 12228 (GenBank Accession ID AE015929) [33]. Positions with less than 10x coverage or less than 90% consensus were excluded from the core genome. An unrooted phylogeny of the population was obtained using the maximum likelihood approximation implemented in FastTree v2.1.5 using the GTR substitution model and both nearest neighbor Interchange (NNI) and subtree pruning and re-grafting (SPR) for hierarchical search methods [34]. iTol v5.5 (https://itol.embl.de) was used for visualization of the phylogeny. Based on pairwise SNP-dissimilarity between isolates, hierarchical clustering was performed using complete-linkage, where clonal groups were defined using a cutoff of 1000 SNPs and categorized according to their most prevalent sequence type.

### Identification of amplicon targets and PCR design

For identification of suitable *S. epidermidis* gene targets for PCR analyses, 50 isolates were selected, representing diverse clusters including commensal lineages and key hospital-associated lineages. The 50 genomes were annotated using Prokka v1.12 [35] and core genes identified using Roary v3.11.2 [36] with default parameters. Core genes were parsed for sequence variability to identify genes in which highly variable regions were flanked by conserved regions (> 95% sequence similarity) making them suitable PCR targets. Using Primer3 v2.3.7 (http://bioinfo.ut.ee/primer3/), all conserved regions flanking a variable region < 1500 bp were analyzed to find potential primers. These candidate primer targets were extracted from all 842 genomes using BLASTN and evaluated based on their potential for discriminating between clonal clusters. No single target sufficiently distinguished all major clusters, therefore two targets were selected; a primary target, *g216* (521 bp), with enough resolution to distinguish between most key clusters – and a secondary target, *yycH* (493 bp), adding resolution to identify additional key clusters. The *yycH*, gene along with *yycL,* functions as a regulator for the expression of the WalK/WalR two-component system which in turn regulates cell wall metabolism through expression of autolysins. Regarding the conserved *g216* gene, it is so far uncharacterized in Staphylococcus species and no information could be found on potential homologues in other species. The primer sequences for amplification of the two targets were: 5′-CGATGCKAAAGTGCCGAATA-3′/5′-CTTCATTTAAGAAGCCACCWTGACT-3′ and 5′-TGGGTATGRCAATCACTTTACA-3′/5′-GCATCAAAAGCACTCTCATTACC-3′ for *yccH* and *g216*, respectively. An unrooted phylogeny of the concatenated gene sequences of the two targets across the population was derived using IQ-TREE v1.6.12 [37] with model selection and 100 bootstrap replicates. Illumina adapter sequences were added to the 5′-end of all the primers to allow for downstream indexing and sequencing.

For determination of the absolute abundance of *S. epidermidis*, a minor groove binder (MGB) probe (TaqMan, Thermo Fisher Scientific) was designed to hybridize to the *g216 S. epidermidis*-specific target (5′-FAM-CATGCCAGATATGAAT-MGB-3′). The MGB probe target sequence was selected based on an alignment of the *g216* gene from all included *S. epidermidis* genome sequences using Qiagen’s CLC Genomics Workbench v11 with subsequent validation in Primer Express v3.0 (Thermo Fisher).

### Collection of primary samples and DNA extraction

For testing of primer specificity, we used DNA from *S. epidermidis* isolates (*n* = 26) representing 15 clusters including major hospital-associated lineages such as ST2, ST5 and ST215, as well as from other staphylococcal species (*n* = 27) and other common skin commensals (*n* = 21), see Supplementary Table 1. Additionally, purified DNA from six *S. epidermidis* isolates with known STs was used to create genomic mock communities in both even and staggered design for method validation. The even mock contained equal copy numbers (10,000) of ST2, ST5, ST14, ST87, ST215, and ST218, whereas the staggered mock contained 10,000 copies of ST87 and ST215, 1000 copies of ST5 and ST14 and 100 copies of ST2 and ST218.

From the isolates, DNA was extracted using an enzymatic pre-lysis step followed by extraction using the Qiagen Blood and Tissue kit (Qiagen). The pre-lysis step was carried out by dissolving bacterial cultures in a lysis buffer containing 20 nM Tris, 2 nM EDTA, 1.2% Triton X and PCR Grade water. Enzymes were added to reach a final concentration of 20 μg/μL lysozyme and 5.56 μg/mL lysostaphin. The enzymatic reaction was incubated for 30 min at 37 °C before processing with the Qiagen Blood and Tissue protocol. DNA concentrations were measured using either Qubit dsDNA BR Assay Kit or Qubit dsDNA HS Assay Kit (Thermo Fisher Scientific).

Primary samples were obtained from 11 healthy community dwelling individuals in Copenhagen, Denmark, from both nose and skin using ESwabs (Copan). Samples from the nares were collected by rotating the swab in one of the anterior nares three times, while skin samples were collected by rubbing the swab against the skin of the participant’s arm (either the volar forearm or antecubital fossa) for 30s. All samples were stored immediately at − 80 °C before processing. DNA was purified from the 22 primary samples, as well as from two blank controls using an enzymatic pre-lysis step before automated purification on a MagNA Pure 96 (Roche Diagnostics A/S) using the MagNA Pure 96 DNA and Viral NA Small Volume Kit and the DNA Blood ds SV 2.0 protocol (Roche Diagnostics A/S). The pre-lysis step was carried out using 200 μL ESwab sample. Enzymes were added to reach a final concentration of 8.4 mol/L of lysozyme, 0.001 U/μL lysostaphin and 0.1 U/μL mytanolysin in 50 μl TE buffer. The enzymatic reaction was incubated for 30 min at 37 °C before addition of 20 μL Proteinase K followed by a 30 min incubation step at 56 °C. All primary samples were purified in technical duplicates to allow subsequent assessment of reproducibility.

### PCR and sequencing

The duplex amplification PCR was carried out using 2x KAPA HiFi (Kapa Biosystems) with 0.2 μM of each primer and 10 μL template in a final volume of 25 μL on a 2720 Thermal Cycler (Applied Biosystems) using the following PCR conditions: 3 min at 95 °C, 25 cycles of 20s at 98 °C, 15 s at 60 °C and 45 s at 72 °C with a final extension step of 5 min at 72 °C. The indexing PCR was carried out using 2x KAPA HiFi, Nextera XT DNA Library Prep Kit v2 for indexing (Illumina) and with 2 μL amplicon product from the amplification PCR in final volume of 25 μL on a 2720 Thermal Cycler (Applied Biosystems) using the following conditions: 3 min at 95 °C, 20 cycles of 20s at 98 °C, 15 s at 55 °C and 45 s at 72 °C, and final 5 min elongation step at 72 °C. After indexing, post-PCR cleanup was performed using a 1:1 ratio of AMPure XP beads (Beckman Coulter) following manufacturer’s instructions before quantification using AccuClear Ultra High Sensitivity dsDNA Quantification Kit (Biotium) following manufacturer’s instructions. Subsequently, samples were pooled in equimolar concentrations and quantified using the Qubit dsDNA HS assay kit (Thermo Fisher Scientific) prior to sequencing on a MiSeq with a 600-cycle MiSeq Reagent Kit v3 (Illumina) and a pool of libraries loaded at 10 pM final concentrations.

### Absolute abundance using qPCR

The *S. epidermidis*-specific *g216* gene was used as basis for a qPCR setup. A standard curve ranging from 1000,000 to 1 copies/μL was generated using AMPure XP bead-purified (× 0,8 ratio, Beckman Coulter) *g216* amplicon generated as described above on DNA purified from an *S. epidermidis* ST2 isolate. The qPCR was performed on a QuantStudio 5 Real-Time PCR system (Thermo Fisher) using PerfeCTA qPCR ToughMix Low ROX (Quantabio) with a 0.5 μM concentration of *g216* primers, 0.15 μM of the *g216* MGB probe, an additional 2 mM MgCl_2_, and 5 μL template in a total volume of 30 μL. qPCR conditions were as follows: 3 min at 95 °C, followed by 40 cycles of 30s at 95 °C, 60s at 60 °C and 60s at 72 °C, with a final 5 min extension step at 72 °C.

### Epidome target gene sequence pre-processing

We used the bcl2fastq Conversion Software (Illumina) to demultiplex raw sequence reads, generating one forward and one reverse FASTQ file for each sample with primer trimming using the Cutadapt software v2.3 [38]. The sequences were processed twice, i.e. the respective primer pairs for each of the two targets were trimmed separately (https://github.com/ssi-dk/epidome/tree/master/scripts). Primer sequences in each read were matched at a tolerated maximum error rate of 6% which corresponds to one mismatch using Cutadapt. To keep a read pair, primers also had to be found in both reads. In order to infer amplicon sequence variants (ASVs) from the trimmed reads, the R package DADA2 v1.12.1 was used [39]. The truncation lengths were adjusted to the expected amplicon lengths of the two target genes, i.e. to obtain ASVs based on the *g216* gene, forward reads were truncated at 276 bp, and reverse reads at 237 bp, aiming at a total length of 513 bp corresponding to an amplicon length of 493 bp with 20 bp overlap for merging. Similarly, for ASV inference based on the *yycH* gene, forward reads were truncated at 278 bp, and reverse reads at 262 bp, aiming at a total length of 540 bp (amplicon length of 520 bp with 20 bp overlap to allow merging). Accordingly, the data was processed with DADA2 separately for each target with default settings except truncation lengths (https://github.com/ssi-dk/epidome/tree/master/scripts). We used the *removeBimeraDenovo* function (method “consensus”) to identify chimeras on a per-sample basis and subsequently removed them from all samples globally. An overview of the per-sample read counts after each filtering step can be found at https://github.com/ssi-dk/epidome/tree/master/example_data.

### Taxonomic database setup and classification

Custom databases of all unique *g216* and *yycH* target sequences can be found at https://github.com/ssi-dk/epidome/tree/master/DB. We formatted our *g216* and *yycH* gene databases to be compatible with DADA2’s *assignTaxonomy* function and used it to classify the *S. epidermidis* ASVs with the RDP naive Bayesian classifier method (https://github.com/ssi-dk/epidome/tree/master/scripts).

ST classification of samples was performed using the *g216* target sequence as the primary identifier. All *g216* sequences unique to a single clonal cluster in the database were immediately classified as the matching clone, and in cases were the *g216* sequence matched multiple clones, the secondary *yycH* target sequences were parsed to determine which clone was present. When this classification failed to resolve due to multiple potential combinations of sequences, ASVs were categorized as “Unclassified”. Similarly, *g216* sequences not found in the database were labelled as “Novel”. The resulting taxonomic count tables are provided at https://github.com/ssi-dk/epidome/tree/master/example_data.

### Statistical analyses

Statistical analyses and generation of graphs were performed in R v3.6.0 (R Core Team 2019). All R scripts documenting the analyses are provided at https://github.com/ssi-dk/epidome/tree/master/scripts. Rarefaction curves were generated by utilizing the R packages phyloseq and ranacapa v0.1.0 [40, 41], and plots were generated with ggplot2 v3.3.1 [42]. The qPCR data was analyzed using QuantStudio Design and Analysis Software (Thermo Fisher). The scatterplot examining correlation between read counts from the two primer pairs was created with ggplot2 v3.3.1 and correlation was tested using Pearson’s correlation. Presentations of the relative abundances of clinical and mock samples were presented in barplots created with ggplot2 v3.3.1 and principal component analysis (PCA) was performed on Euclidean distances between samples based on relative abundances of ASVs. Comparisons between groups were made using ANOSIM tests with 1000 permutations based on the Euclidean distances.

## Supplementary information

Additional file 1: **Supplementary Figure 1.** Read counts after trimming and chimera filtering of the *g216* and *yych* gene targets across all analyzed primary samples. The analyses revealed a higher level of *yycH* read counts compared to *g216,* however with an overall even distribution of across all samples.

Additional file 2: **Supplementary Figure 2.** Rarefaction curves to investigate the dependency of *Staphylococcus epidermidis* abundance/lineages richness on sample library size in mock communities and primary samples. The generated rarefaction curves display the number of observed ASVs over the library size per sample after quality filtering, for *g216* and *yycH* in panel A and B, respectively.

Additional file 3: **Supplementary Figure 3.** Beta-diversity of the *Staphylococcus epidermidis* population across sample sites. Principal component analysis depicting the beta-diversity across all primary samples from skin and nares indicate overlapping populations highlighting samples sites compared to Fig. 4b.

Additional file 4: **Supplementary Table 1.** Bacterial isolates used for laboratory validation of the method. Included are sequence types for all *Staphylococcus epidermidis* isolates, as well as a total of 48 different bacterial species commonly identified as skin commensals that were used for specificity testing; 27 staphylococcal species and 21 non-staphylococcal species.

Additional file 5: **Supplementary Table 2.** qPCR results on standards, as well as primary skin and nasal samples. Presented are the threshold cycle (Ct) values with standard deviation as well as equivalent counts.

## Data Availability

The amplicon sequences are available through the European Nucleotide Archive (ENA) at the European Bioinformatics Institute (EBI) (PRJEB39895). The R scripts for the sequence pre-processing and taxonomy assignment, including the resulting datasets, as well as our custom data bases and R scripts for statistical analyses are available from https://github.com/ssi-dk/epidome/.
