# Supplementary figures and images for: The Epidome - a species-specific approach to assess the population structure and heterogeneity of Staphylococcus epidermidis colonization and infection
Source: BMC Microbiol. 2020 Nov 26;20:362. doi: 10.1186/s12866-020-02041-w (PMC7691061; doi:10.1186/s12866-020-02041-w)

Pearson correlation coefficient: 0.94,  $p = 0.000$

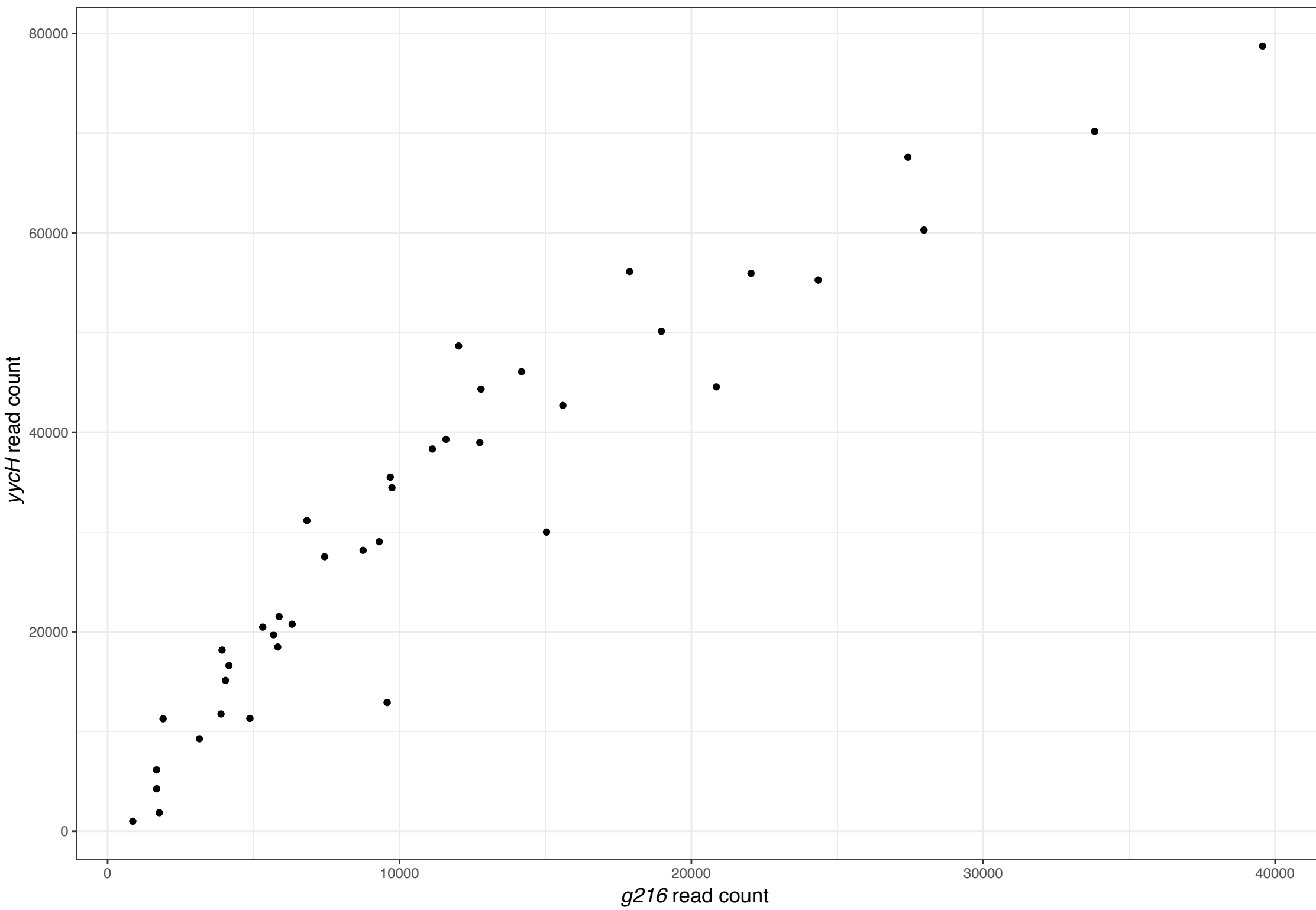

Supplement: Supplementary file 1 — Supplementary Figure 1. Read counts after trimming and chimera filtering of the g216 and yych gene targets across all analyzed primary samples. The analyses revealed a higher level of yycH read counts compared to g216, however with an overall even distribution of across all samples. [file 12866_2020_2041_MOESM1_ESM.pdf]

# Rarefaction curves - *yycH* gene

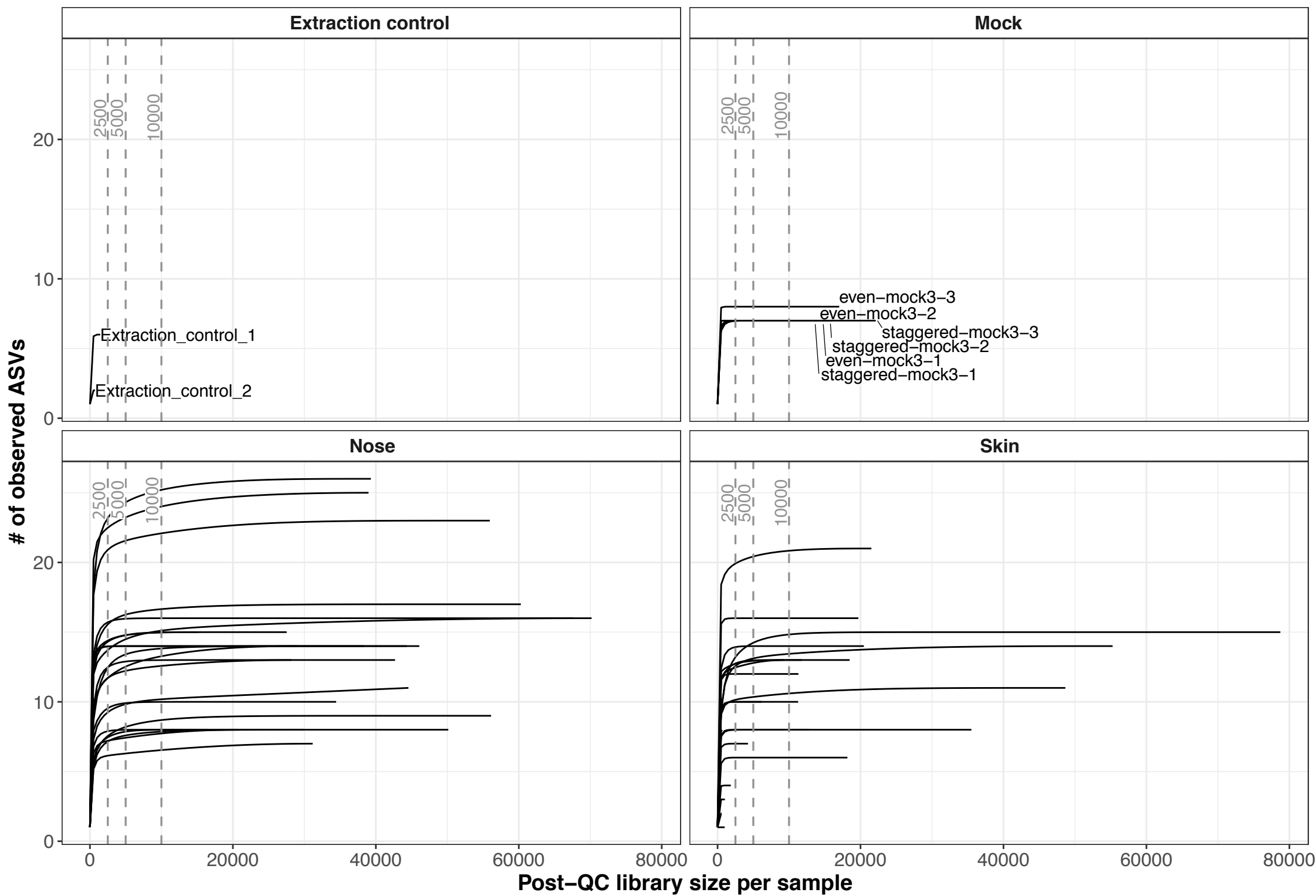

Supplement: Supplementary file 2 — Supplementary Figure 2. Rarefaction curves to investigate the dependency of Staphylococcus epidermidis abundance/lineages richness on sample library size in mock communities and primary samples. The generated rarefaction curves display the number of observed ASVs over the library size per sample after quality filtering, for g216 and yycH in panel A and B, respectively. [file 12866_2020_2041_MOESM2_ESM.zip › Supplementary_Figure_2B.pdf]

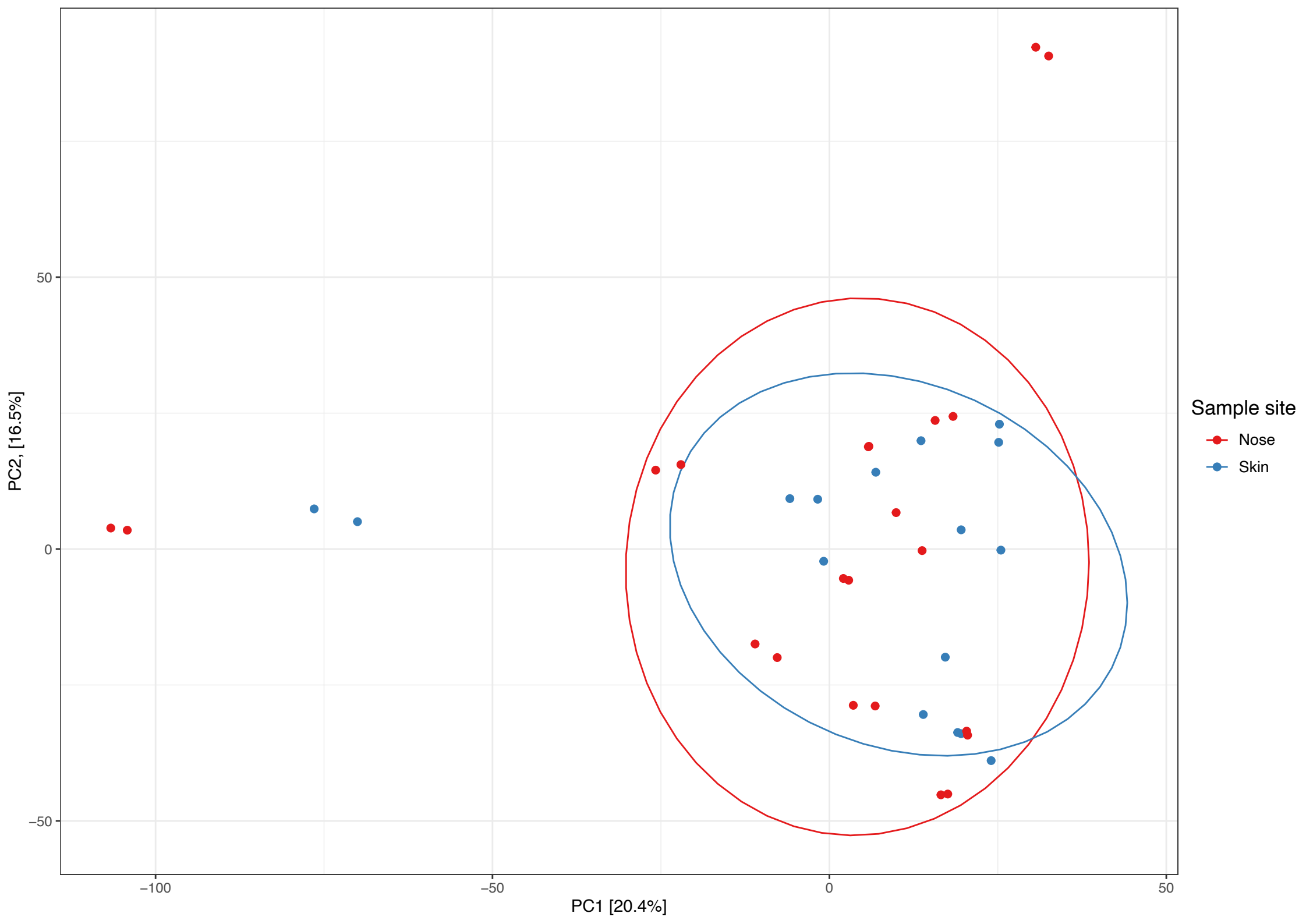

Supplement: Supplementary file 3 — Supplementary Figure 3. Beta-diversity of the Staphylococcus epidermidis population across sample sites. Principal component analysis depicting the beta-diversity across all primary samples from skin and nares indicate overlapping populations highlighting samples sites compared to Fig. 4b. [file 12866_2020_2041_MOESM3_ESM.pdf]
