# Supplementary material for: The Epidome - a species-specific approach to assess the population structure and heterogeneity of Staphylococcus epidermidis colonization and infection
Source: BMC Microbiol. 2020 Nov 26;20:362. doi: 10.1186/s12866-020-02041-w (PMC7691061; doi:10.1186/s12866-020-02041-w)
Supplement: Supplementary file 2 — Supplementary Figure 2. Rarefaction curves to investigate the dependency of Staphylococcus epidermidis abundance/lineages richness on sample library size in mock communities and primary samples. The generated rarefaction curves display the number of observed ASVs over the library size per sample after quality filtering, for g216 and yycH in panel A and B, respectively. [file 12866_2020_2041_MOESM2_ESM.zip › Supplementary_Figure_2A.pdf]

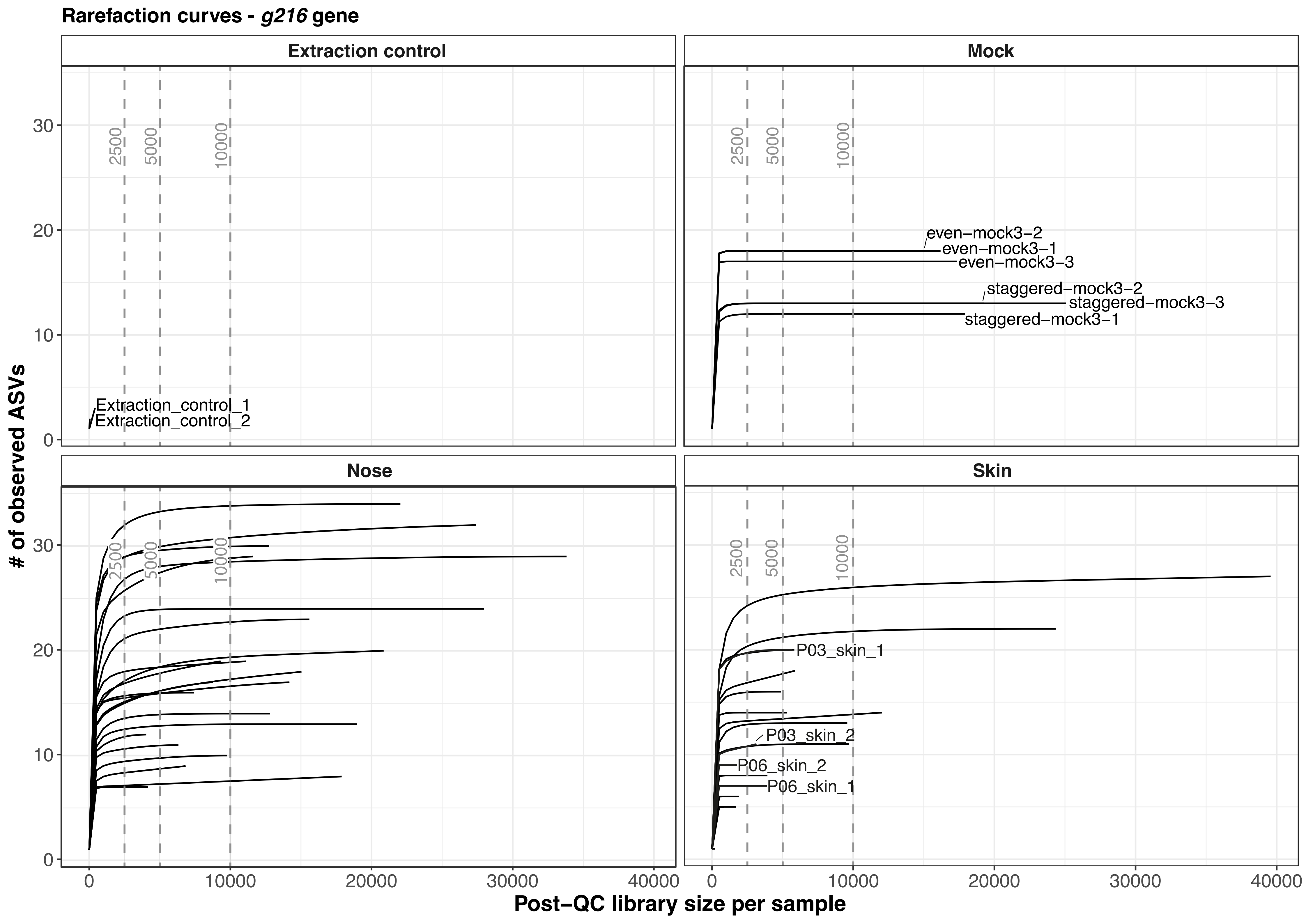

Rarefaction curves - g216 gene
Extraction control
Mock
Nose
Skin
# of observed ASVs
Post−QC library size per sample
2500
5000
10000
Extraction_control_1
Extraction_control_2
even−mock3−2
even−mock3−1
even−mock3−3
staggered−mock3−2
staggered−mock3−3
staggered−mock3−1
P03_skin_1
P03_skin_2
P06_skin_2
P06_skin_1
0
10
20
30
0
10000
20000
30000
40000
